# 3D Framework Carbon for High-Performance Zinc-Ion Capacitors

**DOI:** 10.3390/mi14071476

**Published:** 2023-07-23

**Authors:** Setthathon Kiatikajornjumroen, Xiaopeng Liu, Yinan Lu, Buddha Deka Boruah

**Affiliations:** Institute for Materials Discovery (IMD), University College London (UCL), London WC1E 7JE, UK; setthathon.kiatikajornjumroen.21@alumni.ucl.ac.uk (S.K.); xiaopeng.liu.21@ucl.ac.uk (X.L.); yinan.lu.18@ucl.ac.uk (Y.L.)

**Keywords:** pyrolysis, porous carbon, zinc-ion capacitor

## Abstract

Given the rapid progress and widespread adoption of advanced energy storage devices, there has been a growing interest in aqueous capacitors that offer non-flammable properties and high safety standards. Consequently, extensive research efforts have been dedicated to investigating zinc anodes and low-cost carbonaceous cathode materials. Despite these efforts, the development of high-performance zinc-ion capacitors (ZICs) still faces challenges, such as limited cycling stability and low energy densities. In this study, we present a novel approach to address these challenges. We introduce a three-dimensional (3D) conductive porous carbon framework cathode combined with zinc anode cells, which exhibit exceptional stability and durability in ZICs. Our experimental results reveal remarkable cycling performance, with a capacity retention of approximately 97.3% and a coulombic efficiency of nearly 100% even after 10,000 charge–discharge cycles. These findings represent significant progress in improving the performance of ZICs.

## 1. Introduction

The development of affordable and long-lasting rechargeable energy storage devices is crucial for the advancement of a sustainable economy [1]. These devices are essential for storing intermittent renewable energy, such as solar and wind power, to be used at a later time [2,3,4]. While lithium-ion batteries have found widespread use in electronic mobile devices and electric vehicles [5], their flammable electrolytes make them unsuitable for environmentally safe energy storage applications [6,7]. Aqueous zinc-ion batteries have emerged as a promising option for large-scale energy storage; however, they still face challenges related to low power density and cycle stability [8]. Among the various alternatives, aqueous zinc-ion capacitors (ZICs) have gained significant attention due to their ability to bridge the gap between the high energy density of batteries and the high power density of supercapacitors (SCs) [9,10]. Zinc metal, commonly used as an anode material in ZICs, exhibits a high theoretical gravimetric/volumetric capacity (823 mAh g^−1^; 5851 mAh cm^−3^) and a low redox potential (−0.76 V) [11,12]. Moreover, the use of aqueous electrolytes in ZICs enhances safety and reduces costs [13]. These advantages make ZICs a promising technology for various real-world applications. However, the widespread commercialization of ZICs faces obstacles related to the high production costs and the limited cycling stability of porous carbon cathodes, which significantly impact the energy and power characteristics of ZICs [14,15]. Addressing these challenges is crucial to unlock the full potential of ZICs in practical applications.

Significant progress has been made in the development of porous carbon materials to achieve high-performance ZICs. Various successful carbon electrodes have been engineered using different synthesis approaches. For instance, Shang et al. employed a urea-mediated foaming strategy to synthesize nitrogen-enriched mesoporous carbon nanosheet (NPCN) cathodes. This approach enhanced conductivity, leading to increased charge transfer efficiency and reduced side reactions during zincation [16]. Deng and colleagues reported a N, O co-doped hierarchical porous carbon (HPC) cathode for ZICs, exhibiting a high specific capacity of 138.5 mA h g^−1^ and excellent cycle lifetime with no capacity decay after 10,000 cycles [17]. Other researchers have explored various carbon morphologies, such as graphene and graphite, to achieve low-entropy carbonaceous structures with improved crystallinity, resulting in enhanced life-cycling stability [18,19,20,21]. However, many of these processes involve complex steps, high temperatures, and the use of toxic chemicals, which increase the cost of electrode powder preparation [22,23]. Hence, there is an urgent need to explore simple and cost-effective methods for synthesizing porous carbon cathodes to enable practical applications of ZICs.

In this study, we obtained a three-dimensional (3D) conductive porous carbon framework cathode through the direct pyrolysis of sodium citrate. The resulting porosity structure of the electrode facilitates ion diffusion, leading to excellent rate capability. Furthermore, the porous carbon framework exhibits superior cycling stability, maintaining a capacity of 97.3% after 10,000 cycles at 10 A g^−1^ and demonstrating excellent reversible rate capability with a higher recovery rate.

## 2. Materials and Methods

Porous Carbon Derivation: To prepare the porous carbon, the procedure involves placing sodium citrate in the alumina sample container, which is then inserted into the quartz tube of an Elite Thermal Systems Limited controlled atmospheric furnace (Eurotherm-3216-3208-3204 PID Built-in). To ensure precise temperature control, a two-step process is implemented. Instead of directly setting the temperature to 650 °C, which could cause overshooting, we take precautions. In step 1, we heat the furnace to 500 °C and maintain it for 30 min. This helps stabilize the temperature and prevents it from exceeding 650 °C. In step 2, we gradually increase the temperature to 650 °C and hold it constant for 1 h before cooling it down. Before heating, argon gas is purged into the quartz tube at 2 PSI for 30 min to create an argon atmosphere and reduce oxygen content.

Once the sodium citrate is fully pyrolyzed and cooled to room temperature, the remaining sample consists of tar, charcoal, and sodium-based salts (Na_2_CO_3_). These samples are transferred to a beaker and filled with deionized water. The beaker is then heated on a heater plate inside a fume hood at 80 °C for 30 min to remove the salts from the porous carbon. Subsequently, a vacuum filtration setup is created using Whatman filter paper to capture the porous carbon. The warm solution in the beaker is slowly poured into the vacuum filtration apparatus. Once the filtration process is complete, ethanol solvent is added to rinse the equipment, followed by additional warm water rinses for three cycles to remove any residual salts. The samples on the Whatman filter paper are then placed in a glass petri dish and left overnight in a vacuum furnace set to 100 °C. Finally, the obtained porous carbon is transferred to the vessel of a Thinky Mixer Model ARM-310 along with six zirconia balls (6.4987 g). The mixer operates at 2000 RPM for 30 min, ensuring proper mixing and homogeneity.

Material Characterization: The powder samples’ morphology was examined using Oxford Instrument Wave ZEISS Scanning Electron Microscopy (SEM). To determine the lattice and phase structure of the powder samples, X-ray diffraction (XRD) analysis was conducted using AERIS PANalytical with a Cu Ka X-ray source. The XRD measurements spanned an angle range of 10° to 80°. The bonds present in the porous carbon were investigated using Fourier-Transform Infrared Spectroscopy (FTIR) with a Perkin-Elmer instrument. Additionally, the N_2_ adsorption–desorption isotherms of the samples were collected utilizing a Quantachrome instrument to evaluate the surface area and porosity of the samples. Furthermore, Raman spectra of the synthesized samples were obtained using a Renishaw instrument, providing valuable insights into the carbonaceous structure.

Cell Assembly: The cathode slurry was prepared by combining all the active materials, Super P Conductive, and PVDF binder at a weight ratio of 70 wt%:20 wt%:10 wt% using a Thinky Mixer Model ARM-310. Initially, the active materials were mixed with Super P, followed by the addition of a specific amount of PVDF dissolved in NMP (N-Methyl-2-pyrrolidone). The mixture was thoroughly blended to achieve a homogeneous slurry. Subsequently, the homogenized slurry was drop-cast onto carbon felt current collectors and dried overnight at 70 °C in a vacuum oven. After drying, the cells were assembled in CR2032 coin cell configuration. The assembly involved placing a 0.25 mm zinc anode foil in contact with the porous carbon electrode, with a glass fiber Whatman porous separator in between. The electrolyte used was 3 M Zn(CF_3_SO_3_)_2_ in an aqueous solution. The cell assembly was carried out in an open atmosphere. To ensure proper conditioning, the cells were left undisturbed for 10 h before undergoing electrochemical characterization.

Electrochemical Performance: The electrochemical performance of the ZIC was evaluated using different instruments. Specifically, Biologic VMP-3, Neware Battery Testing System, and Gramry Instruments Interface 1010E potentiostats were utilized for testing. Cyclic voltammetry (CV) measurements were conducted at various scan rates ranging from 10 to 1000 mV s^−1^. The galvanostatic charge–discharge performances were evaluated at different specific currents, ranging from 0.1 to 20 A g^−1^, over a voltage range of 0.2 to 1.8 V. To assess long-term cycling performance, a Neware Battery Testing System was used to conduct cycling tests at a specific current of 10 A g^−1^ for 10,000 cycles. Electrochemical impedance spectroscopy (EIS) measurements were performed using the EIS Gramry Instruments Interface 1010E Potentiostat/Galvanostat/ZRA 21128. The measurements were carried out in the frequency range of 10 mHz to 100 kHz, with a voltage amplitude of 10 mV. The EIS measurements were conducted using the Gramry Framework program.

## 3. Results

**Figure 1:**
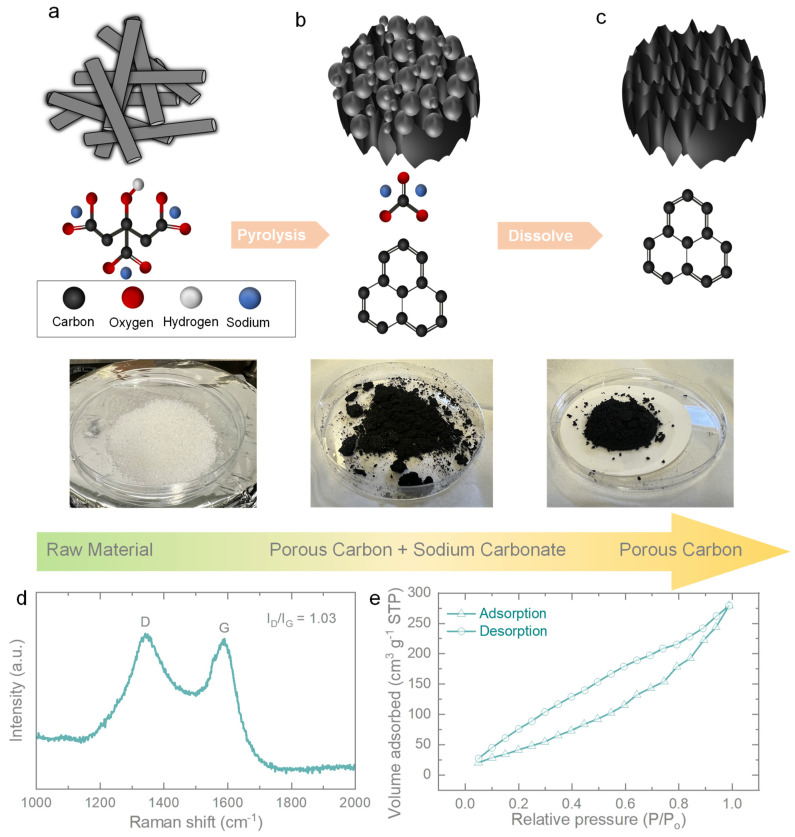
Schematic representing (**a**–**c**) diagrams of porous molecular and structural transformations that cause the pyrolyzed sodium citrate to form porous carbon as a cathode. The below images are digital photographs of the raw material, pyrolyzed sodium citrate, and porous carbon. (**d**,**e**) Raman and N_2_ adsorption–desorption isotherms of porous carbon.

Figure 1a–c illustrate the schematic synthesis procedure for the direct pyrolysis of sodium citrate. Through a self-assembly process, porous carbon is obtained. In this process, sodium citrate is directly heated in a tube furnace, gradually decomposing and leaving behind carbon within the structure. The carbon particles self-assemble, leading to the formation of porous carbon. Figure 1d presents the Raman spectrum of the porous carbon. The characteristic peaks of amorphous carbon, namely, the D-band (associated with disorder) and G-band (related to graphitic carbon), are observed at around 1345 and 1580 cm^−1^ [24]. The intensity ratio (I_D_/I_G_) is measured to be 1.03, indicating an acceptable degree of graphitization for the prepared porous carbon. The D-band is attributed to sp3-hybridized carbon, while the G-band corresponds to ordered graphitic sp2-hybridized carbon [25]. To assess the surface area and porous structure of the porous carbon, N_2_ adsorption–desorption measurements were conducted. Figure 1e presents a typical N_2_ adsorption–desorption isotherm, and the specific surface area of the as-prepared porous carbon was measured to be 194.65 m^2^ g^−1^.

**Figure 2:**
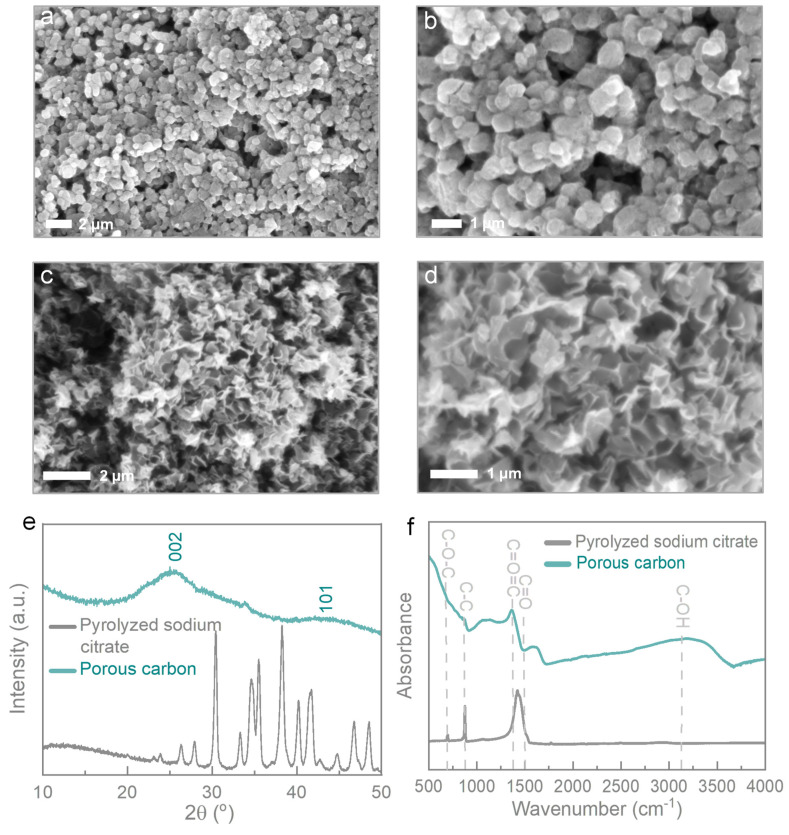
Low- and high-magnification SEM images of (**a**,**b**) sodium citrate and (**c**,**d**) porous carbon. (**e**,**f**) XRD patterns and FTIR curves for pyrolyzed sodium citrate and porous carbon, respectively.

Figure 2a,b present scanning electron microscopy (SEM) images of the sodium citrate samples. Upon dissolving the as-pyrolyzed sodium citrate, the generation of a 3D porous carbon structure is observed (Figure 2c,d). Figure 2e provides the XRD patterns of the as-prepared sodium citrate and porous carbon. The characteristic peaks observed in the XRD patterns correspond to the lattice planes of sodium carbonate material, specifically (002), (310), (112), and (400), which are in accordance with JCPDS card no. 72-0628 [26]. The peaks at 2θ = 26° and 43° correspond to the lattice planes of carbon material (002) and (101), respectively, according to JCPDS no. 41-1487. These peaks indicate the presence of amorphous carbon embedded within the sodium carbonate [27].

Figure 2f displays the FTIR spectra of pyrolyzed sodium citrate and porous carbon in the range of 4000–500 cm^−1^. The absorption peaks at 695, 879, 1412, 1774, and 3310 cm^−1^ in the pyrolyzed sodium citrate spectrum correspond to the vibrational bonds of C–O–C, C–C, C=O, O=C=O, and C–OH, respectively [28]. These findings confirm the generation of hydrocarbons during and after the pyrolysis process of sodium citrate. The existence of carbonate-based material is evident from the carbon–oxygen peaks in the spectra. The peaks related to the C–C bond indicate the presence of carbon embedded within the sodium carbonate. After dissolving the salt to form porous carbon, the spectrum of porous carbon shows peaks for C–C (carbon) and C–OH (hydroxyl), indicating residue contamination from salt and ethyl alcohol used during the dissolution process. The occurrence of the C–OH group is due to the interaction between water and carbon, resulting in surface modifications and the formation of hydroxyl bonds. The presence of C=O=C peaks indicates interaction with oxygen.

**Figure 3:**
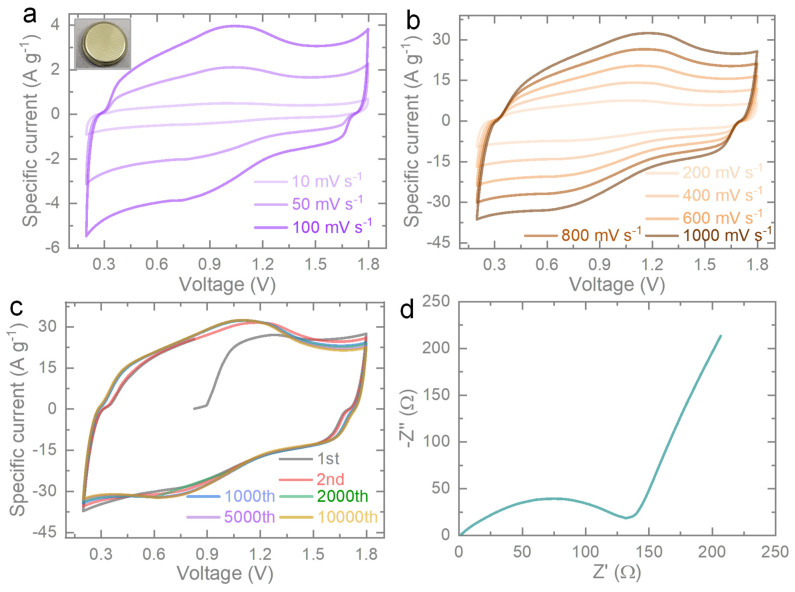
Graphical result for the electrochemical test including CV measurement at (**a**) 10–100 mV s^−1^ (inset demonstrates a digital photograph of a coin-cell-based ZIC) and (**b**) 200–1000 mV s^−1^ in a voltage window 0.2–1.8 V. (**c**) Cycling CVs at 1000 mV s^−1^ for 1st, 2nd, 1000th, 2000th, 5000th, and 10,000th cycles. (**d**) EIS (electrochemical impedance spectroscopy) measurement of the Zn//PC measured in the frequency range of 10 mHz to 100 kHz at a voltage amplitude of 10 mV before cycling.

To depict the charge storage performance of the ZIC, Figure 3a presents Cyclic Voltammetry (CV) measurements conducted over a voltage range of 0.2 to 1.8 V at various scan rates ranging from 10 to 100 mV s^−1^. The CV curves exhibit consistent redox shapes across different scan rates, indicating excellent rate capability even at a high scan rate of 1000 mV s^−1^ (Figure 3b). The rectangular shapes remain well-preserved without significant deformation at various scan rates, demonstrating the robustness of the ZIC system. To verify the stability of the prepared porous carbon, Figure 3c displays the CV curves of the ZIC system at a scan rate of 1000 mV s^−1^ for 10,000 cycles. It is evident that the curves overlap without noticeable changes throughout the 10,000 cycles, indicating the high charge transfer efficiency and exceptional stability of our porous carbon material.

Figure 3d showcases the Nyquist plot of the porous carbon, wherein the semi-circular curves in the high-frequency range represent the charge transfer resistance (Rct) while the slope lines in the low-frequency region correspond to the lower equivalent series resistance (Rs). The ZIC exhibits an Rct value of 130.04 Ω and an Rs value of 2.03 Ω. The electrode demonstrates nearly unimpeded ion diffusion, which is attributed to the 3D porous structure of the material, thereby enhancing its performance.

**Figure 4:**
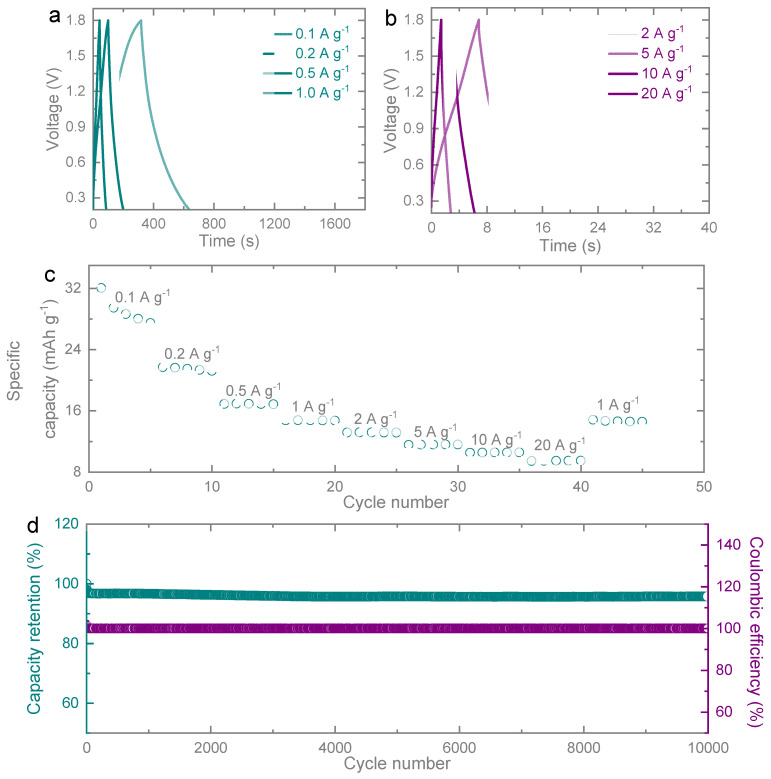
(**a**) Galvanostatic charge–discharge curves at specific currents of (**a**) 0.1–1.0 A g^−1^ and (**b**) 2–20 A g^−1^ recorded in a voltage window of 0.2–1.8 V. (**c**) Rate capability test of the Zn//PC cell at specific currents of 0.1, 0.2, 0.5, 1.0, 2.0, 5.0, 10.0, 20.0, and 1.0 A g^−1^ for 5 cycles at each specific current. (**d**) Graphical results for the electrochemical test including the long-term cycling performance at a current density of 10 A g^−1^ for 10,000 cycles, which illustrates the capacity retention and coulombic efficiency curves.

Figure 4a,b present the galvanostatic charge–discharge curves of the ZIC. The observed high symmetry between the anodic charging and cathodic discharging segments in the galvanostatic charge–discharge curve indicates the presence of a highly reversible capacitive material. This symmetry confirms the excellent reversibility of the ZIC system. Furthermore, the capacitance retention remains favorable even when the current density reaches a maximum of 20 A g^−1^. However, at higher current densities, such as those depicted in Figure 4b, the interaction between diffusion ions and electrodes in the electrolyte becomes more pronounced, leading to a limitation in ion diffusion and a subsequent decrease in specific capacitance.

Figure 4c was utilized to measure the rate capability of the ZIC and assess its energy storage capacity. The specific capacities of the ZIC were measured at different specific currents: 32.05, 21.20, 16.87, 14.72, 13.15, 11.54, 10.51, 9.40, and 14.80 mAh g^−1^ for current densities of 0.1, 0.2, 0.5, 1.0, 2.0, 5.0, 10.0, 20.0, and 1.0 A g^−1^, respectively. Remarkably, when the current density reverted to 1.0 A g-1, a capacity of 14.80 mAh g^−1^ was maintained with a recovery rate of up to 99.46%. This observation demonstrates the excellent reversible rate capability of the porous carbon-based capacitor. Moving on to Figure 4d, our ZIC exhibited stable cycling performance over 10,000 cycles at a specific current of 10 A g^−1^. Throughout these cycles, a capacity retention of 97.3% and a coulombic efficiency close to 100% were achieved. This highlights the ability of the porous carbon cathode to provide highly stable electrochemical reversibility over extended cycling periods. The observed drop in retention capacity percentage can be attributed to the formation of a thin passivation layer on the zinc anode, which hinders the interfacial transportation of zinc ions. Additionally, the presence of dendrites on the zinc anode contributes to the gradual degradation in retention throughout the cycles. Although a slight decrease in specific capacitance was observed during the initial cycles in the ZIC electrodes, the overall trend in cycling performance indicated that porous carbon exhibits superior stability and prolonged cycling life for electrode-specific capacity (as shown in Table 1).

## 4. Conclusions

To summarize, we successfully synthesized a conductive porous carbon framework cathode with three-dimensional (3D) growth. This was achieved through the thermal decomposition of sodium citrate followed by carbonization. The 3D porosity structure of the electrode enables enhanced ion transportation by creating additional channels that reduce transport resistance and minimize diffusion pathways. As a result, our zinc-ion capacitor demonstrated exceptional reversible rate capability, with a recovery rate of 99.46% when transitioning from 20 A g^−1^ to 1 A g^−1^. Furthermore, it exhibited a stable cycling performance, with a capacity retention of 97.3% after 10,000 cycles at 10 A g^−1^. This work presents a single-step synthesis route for producing high-performance porous carbon cathodes specifically designed for ZICs.

## Figures and Tables

**Table 1:** Electrochemical performance of ZICs based on different carbon-based cathode materials reported in recent works.

Device (Cathode//Anode)	Electrolyte	Cyclic Stability %	Reference No.
N-doped porous carbons//Zn	2 M ZnSO_4_	84.9 after 10,000 cycles at 5 A g^−1^	16
HPC/CC//Zn	ZnSO_4_/gelatin gel	114.3 after 10,000 cycles at 5 A g^−1^	17
Chemical activated graphene//Zn	3 M Zn(CF_3_SO_3_)_2_	93 after 80,000 cycles at 8 A g^−1^	18
Rubidium-activated porous carbon//Zn	gelatin/Zn(CF_3_SO_3_)_2_ gel	99.8 after 20,000 cycles at 10 A g^−1^	19
VO_0.9_/C//Zn PC//Zn	3 M Zn(CF_3_SO_3_)_2_ 3 M Zn(CF_3_SO_3_)_2_	84.3 after 10,000 cycles at 3 A g^−1^ 97.3 after 10,000 cycles at 10 A g^−1^	21 This work

## Data Availability

The data used in this study are available on request.
